# Transcriptome Profiling Reveals Transcriptional Regulation by DNA Methyltransferase Inhibitor 5-Aza-2′-Deoxycytidine Enhancing Red Pigmentation in Bagged “Granny Smith” Apples (*Malus domestica*)

**DOI:** 10.3390/ijms19103133

**Published:** 2018-10-12

**Authors:** Changqing Ma, Bowen Liang, Bo Chang, Li Liu, Jiuying Yan, Yazhou Yang, Zhengyang Zhao

**Affiliations:** 1 State Key Laboratory of Crop Stress Biology for Arid Areas, College of Horticulture, Northwest A & F University, Yangling 712100, China; macqing@126.com (C.M.); lbwnwsuaf@126.com (B.L.); cbl9132322@163.com (B.C.); li91761092@163.com (L.L.); yanjiuying123@163.com (J.Y.); youngasia@126.com (Y.Y.); 2 Shaanxi Research Center of Apple Engineering and Technology, Yangling 712100, China

**Keywords:** Granny Smith, anthocyanin, transcriptome, 5-aza-dC, *MYB*, DNA methylation

## Abstract

The red color of apples (*Malus domestica*) is an attractive trait for consumers. The green skinned “Granny Smith” cultivar develops red pigmentation after bagging treatment. DNA methylation plays an important role in various developmental processes in plants. To explore the possible functions of DNA methylation in the pigmentation of bagged “Granny Smith” apples, we first analyzed the anthocyanin content of fruit skin following treatment with the DNA methyltransferase inhibitor 5-aza-2′-deoxycytidine (5-aza-dC). The results revealed an increase in anthocyanin content in bagged fruits following 5-aza-dC treatment, while no anthocyanins were detected in unbagged fruits. In addition, 8482 differentially expressed genes between 5-aza-dC-treated and control groups were identified in bagged fruits by RNA sequencing, including genes encoding transcription factors, enzymes related to anthocyanin accumulation, and methylases. Changes in the expression of these genes may be responsible for 5-aza-dC-induced red pigmentation in bagged fruits of “Granny Smith”. The findings provide novel evidence for the involvement of DNA methylation in the red pigmentation of non-red-skinned apples.

## 1. Introduction

Apple (*Malus domestica*) fruits are popular among consumers and an important source of nutrients [1]. The red color of apple skin is attributed to the presence of anthocyanins [2]. Anthocyanins comprise a major class of flavonoid pigments and represent an excellent source of antioxidants that contribute to human health [3,4,5]. Thus, the abundance of anthocyanins in apple skin affects consumer choice and marketability [6]. The major anthocyanin pigment present in apple skin is cyanidin 3-galactoside [7].

The structural genes directly involved in anthocyanin biosynthesis in apples have been well characterized, and include genes encoding phenylalanine ammonialyase (PAL), 4-coumarate coenzyme A ligase (4CL), chalcone synthase (CHS), chalcone isomerase (CHI), flavanone 3-hydroxylase (F3H), dihydroflavonol 4-reductase (DFR), leucoanthocyanidin dioxygenase (LDOX), and UDP-glucose:flavonoid 3-O-glucosyltransferase (UFGT) [8,9]. The expression of these structural genes is regulated at the transcriptional level by the MYB-bHLH-WD40 (MBW) complex, which includes R2R3-MYB, basic helix-loop-helix (bHLH), and WD40 components [10,11]. Additionally, anthocyanin biosynthesis is regulated by other transcription factors (TFs) such as NAM/ATAF/CUC (NAC) and protein domain containing a tryptophan-arginine-lysine-tyrosine amino acid motif (WRKY) family members [12,13]. Currently, little is known about how TF expression is regulated in the process of red skin pigmentation in non-red apple cultivars.

DNA methylation is a prominent epigenetic modification in eukaryote genomes, and it has been linked to fruit color. For example, in two *Oncidium* orchid cultivars, differences in DNA methylation status affect *CHS*-mediated anthocyanin pigmentation in floral tissues [14]. Moreover, compared with the red-skinned Max Red Bartlett pear (*Pyrus communis*), increased methylation of the *PcMYB* promoter and reduced expression of *PcMYB* were reported in a green-skin phenotype [15]. Similarly, in “Honeycrisp” apples, the red striped fruits display lower overall methylation levels throughout the promoter region of *MYB* than green striped fruits [16]. In addition, bagging treatment negatively regulates DNA methylation of the *MYB* promoter, resulting in red pigmentation in the non-red skinned apple cultivars such as “Granny Smith” and “Mutsu” [17,18]. However, the mechanisms of DNA methylation responsible for red skin pigmentation in non-red apple cultivars have not been well documented.

Cytosine methylation is regulated by DNA methyltransferases (methyltransferase (MET) and DNA (cytosine-5)-methyltransferase (DRM)) [19] and demethylases (demethylase (DME) and repressor of gene silencing (ROS)) [20]. The proper regulation of cytosine methylation is essential for maintaining chromatin structure and retarding appropriate gene expression [21,22]. 5-Aza-2′-deoxycytidine (5-aza-dC) is a strong inhibitor of DNA methylation that can irreversibly bind methyltransferase enzymes when incorporated into DNA. Exposure of plants to exogenous 5-aza-dC can induce phenotypic trait variation by restricting DNA methylation. A range of plant species have shown this phenomenon, such as dwarfism in rice (*Oryza sativa*) [23], early flowering in *Arabidopsis* [24] and strawberry (*Fragaria vesca*) [25], and abnormal flower development and leaf morphology in potato (*Solanum ruiz-lealii*) [26].

“Granny Smith” is a green-skinned apple, which can turn cardinal red after bagging treatment during fruit ripening in the Loess Plateau region of China [27,28]. In a recent study, we found that 5-aza-dC treatment induced anthocyanin biosynthesis in “Granny Smith” [17]. The present study was undertaken to further explore the mechanisms of red pigmentation in bagged fruits of “Granny Smith” following 5-aza-dC treatment. We performed RNA-seq analysis to compare the transcriptomes of “Granny Smith” skins with and without 5-aza-dC treatment after bagging. The results revealed that numerous genes were differentially expressed, including genes encoding TFs, anthocyanin accumulation-related enzymes, and methylases. These findings provide insight into differential gene expression in the response to 5-aza-dC (demethylation) treatment and improve our understanding of the regulatory mechanisms associated with the red pigmentation of “Granny Smith” apples and possibly other non-red apple fruits.

## 2. Results

### 2.1. Changes in Red Pigmentation Patterns in the Skin of “Granny Smith” Apples

**Figure 1:**
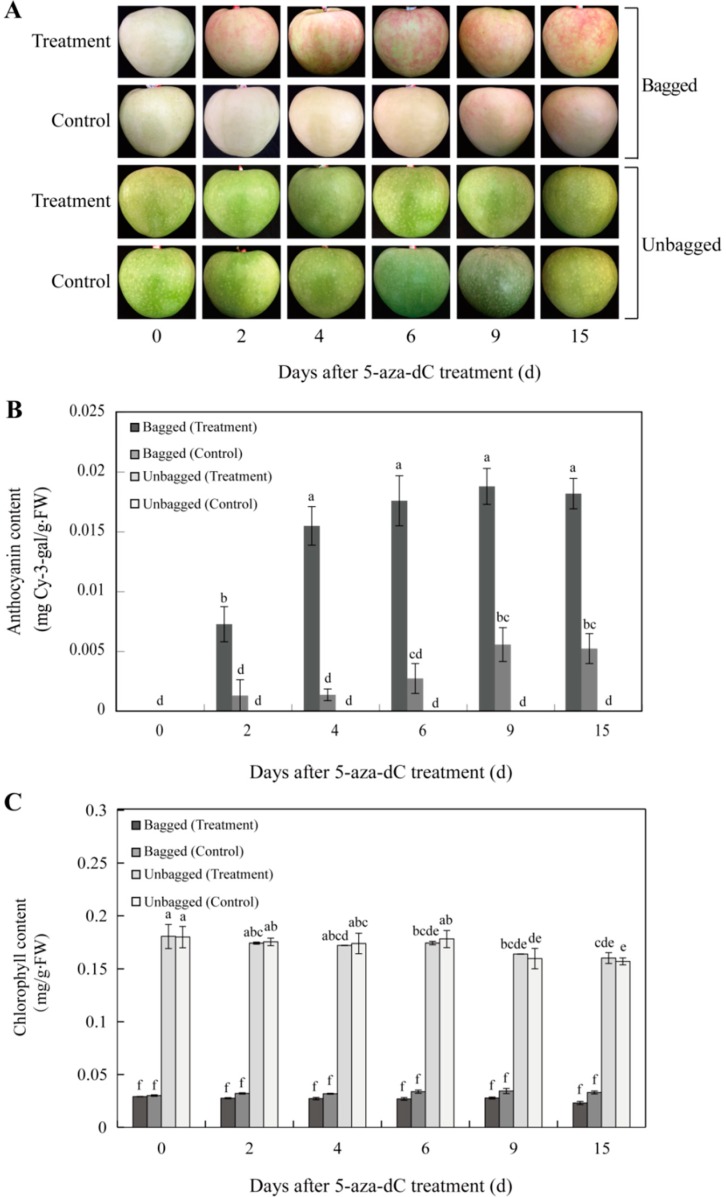
Effect of 5-aza-2′-deoxycytidine (5-aza-dC) treatment on skin color and pigmentation in “Granny Smith” apples. (**A**) Changes in skin color. (**B**) Dynamic characteristics of cyanidin3-galactoside and (**C**) differences in chlorophyll content. Error bars indicate standard deviation (SD) obtained from four biological replicates. Different lowercase letters indicate significant differences between the treated and control groups of bagged or unbagged fruits by Tukey’s multiple range test (*p* < 0.05).

Compared with their respective control fruits, bagged fruits gradually turned red in color during the 5-aza-dC treatment, whereas unbagged fruits did not change color (Figure 1A). When 5-aza-dC-treated apples were exposed to light, the anthocyanin contents in the skins of bagged fruits increased and were significantly higher in treated fruits than in the control fruits. However, in unbagged fruits, no anthocyanins were detected in either of the two groups during the time-course of 5-aza-dC treatment (Figure 1B). Additionally, the chlorophyll content did not significantly differ between the treated and control groups of bagged or unbagged fruits (Figure 1C). These results suggest that 5-aza-dC induced anthocyanin biosynthesis in bagged apple skin.

### 2.2. RNA-Seq and Annotation Analysis of Differentially Expressed Genes (DEGs)

**Figure 2:**
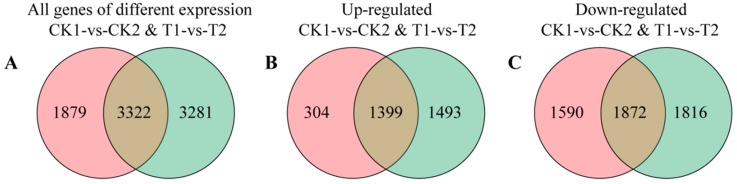
Venn diagrams of all genes and differentially expressed genes (DEGs) in skin samples between 5-aza-dC-treated and control groups of bagged “Granny Smith” apples. (**A**) Intersection of the Venn diagram showing all DEGs identified in pair analyses (CK1 vs. CK2 and T1 vs. T2). (**B**,**C**) Intersection of the Venn diagram showing up- and down-regulated DEGs between pairs, respectively.

To gain global insight into the molecular mechanisms responsible for skin color changes in bagged apples after 5-aza-dC treatment, 5-aza-dC-treated (T) and control (CK) skin samples were collected at 0 days after exposure of 5-aza-dC-treated fruits to light (DAFT, T1 and CK1) and at 15 DAFT (T2 and CK2) for transcriptomic analysis. A statistical summary of RNA-seq results is given in Table 1. The genomic mapping rate was greater than 55.61%, and the gene mapping rate reached 50.33–64.21%. Venn diagrams of all DEGs in skin samples of T and CK groups are shown in Figure 2. In T groups, 2892 DEGs were upregulated and 3688 were downregulated; in CK groups, only 1703 DEGs were upregulated and 3462 were downregulated. Thus, a larger number of genes were altered in T groups than CK groups, suggesting that 5-aza-dC had a major influence on apple fruits.

**Figure 3:**
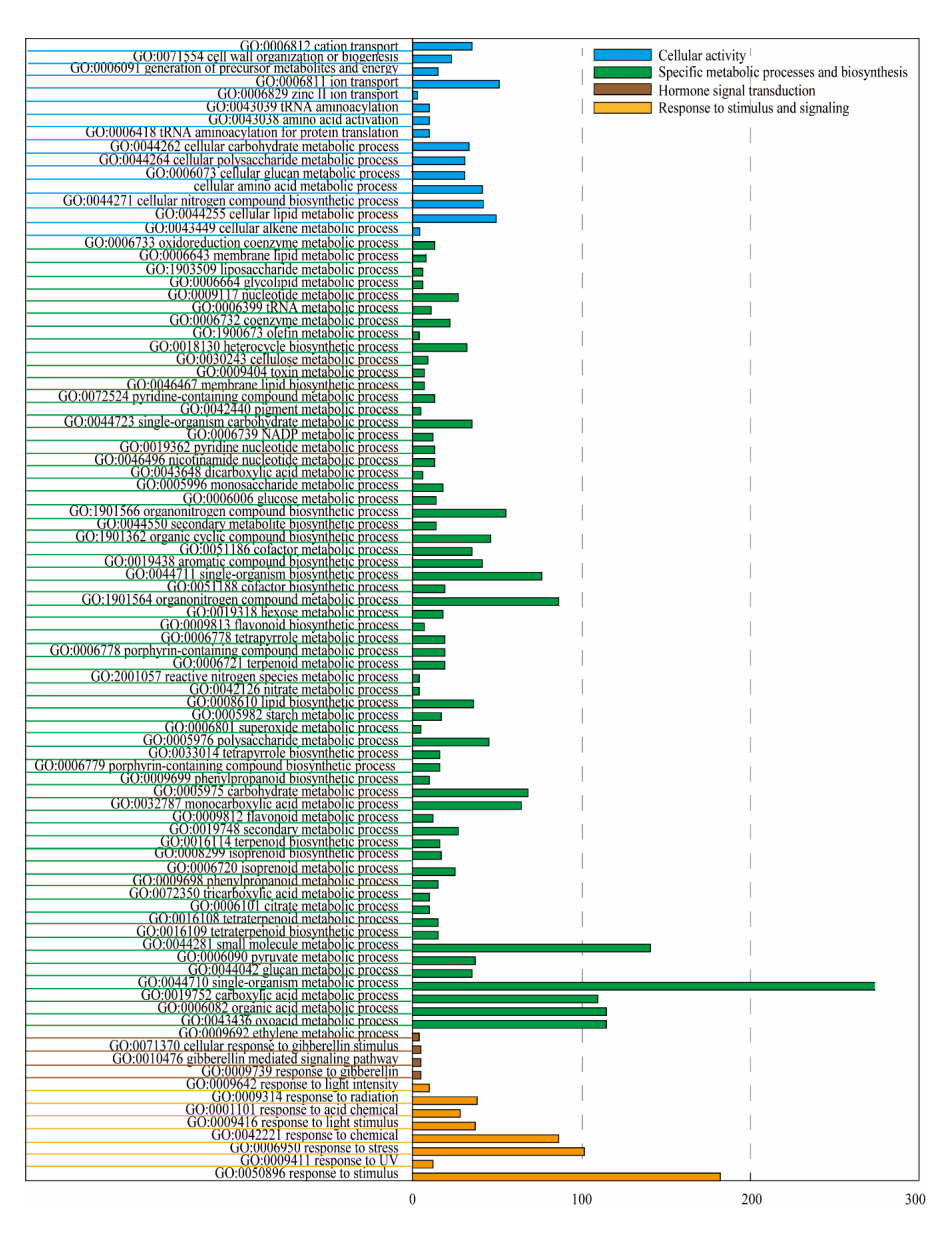
Clusters of annotated Gene Ontology (GO) terms in the “biological process” category enriched in 3281 DEGs in “Granny Smith” apple skins. DEGs were classified into specific “biological process” categories using DAVID (available online: https://david.ncifcrf.gov) with high classification stringency (*p* < 0.05). The horizontal ordinate represents the number of genes in the category.

Gene Ontology (GO) and Kyoto Encyclopedia of Genes and Genomes (KEGG) pathway enrichment analyses were used to respectively identify biological processes and functions enriched in DEGs. Significantly enriched GO biological terms included those in the categories of cellular activity, specific metabolic processes, and biosynthesis, hormone signal transduction, and response to stimulus and signaling (Figure 3). The most heavily enriched KEGG pathways were related to specific metabolic and biosynthetic processes (Table 2). Among these pathways, the most significantly enriched was flavonoid biosynthesis, suggesting that 5-aza-dC had an important effect on apple color.

### 2.3. Expression of Anthocyanin Biosynthesis-Related Genes

**Figure 4:**
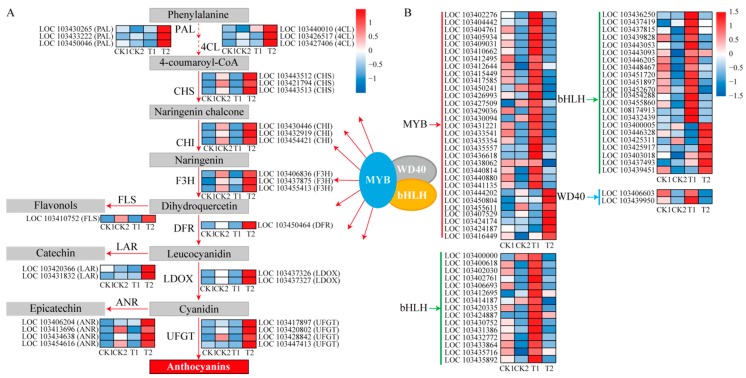
Simplified scheme and heat map of the expression of genes related to anthocyanin biosynthesis in “Granny Smith” apple skins. (**A**) Structural gene expression in “Granny Smith” apple skins. Straight-line and dashed-line arrows indicated continuous and discontinuous steps, respectively. (**B**) Regulatory gene expression in “Granny Smith” apple skins. Arrows with different colors indicated different regulatory genes. PAL, phenylalanine ammonia lyase; 4CL, 4-coumarate coenzyme A ligase; CHS, chalcone synthase; CHI, chalcone isomerase; F3H, flavanone 3-hydroxylase; DFR, dihydroflavonol-4-reductase; LDOX, leucoanthocyanidin dioxygenase; UFGT, UDP-glucose: flavonoid-3-*O*-glucosyltransferase; FLS, flavonol synthase; LAR, leucoanthocyanidin reductase; ANR, anthocyanidin reductase. Enzyme names, gene IDs, and expression patterns are indicated at the side of each step. The color scale on the right represents the log-transformed Fragments Per Kilobase of transcript per Million (FPKM) value.

In order to explore the effect of 5-aza-dC on red skin pigmentation in “Granny Smith” apples, structural and regulatory genes involved in anthocyanin biosynthesis were identified (Figure 4). Transcriptional levels of structural genes, such as *PAL*, *4CL*, *CHS*, *CHI*, *F3H*, *DFR*, *LDOX*, and *UFGT*, were increased in response to 5-aza-dC treatment. In particular, transcription levels of three *PALs* (LOC103430265, LOC103433222, and LOC103450046), three *4CLs* (LOC103440010, LOC103426517, and LOC103427406), three *CHSs* (LOC103443512, LOC103421794, and LOC103443513), three *CHIs* (LOC103430446, LOC103432919, and LOC103454421), three *F3Hs* (LOC103406836, LOC103437875, and LOC103455413), one *DFR* (LOC103450464), two *LDOXs* (LOC103437326 and LOC103437327) and four *UFGTs* (LOC103417897, LOC103420802, LOC103428842, and LOC103447413) were significantly upregulated in the T2 group. Moreover, transcriptional levels of genes related to other flavonoid metabolic pathways were also significantly increased in T2 group, including one flavonol synthase (*FLS*) gene (LOC103410752), two leucoanthocyanidin reductase (*LAR*) genes (LOC103420366 and LOC103431832), and four anthocyanidin reductase (*ANR*) genes (LOC103406204, LOC103413696, LOC103434638, and LOC103454616; Figure 4A).

In addition, transcription levels of seven *MYB* genes (LOC103444202, LOC103450804, LOC103455611, LOC103407529, LOC103424174, LOC103424187, and LOC103416449) and seven *bHLH* genes (LOC103400005, LOC103446328, LOC103425311, LOC103425917, LOC103403018, LOC103437493, and LOC103439451,) were significantly upregulated in the T2 group, while other candidate *MYB*, *bHLH*, and *WD40* genes were upregulated in the T1 group (Figure 4B).

### 2.4. Transcription Factors Are Differentially Expressed in Response to 5-Aza-dC Treatment

**Figure 5:**
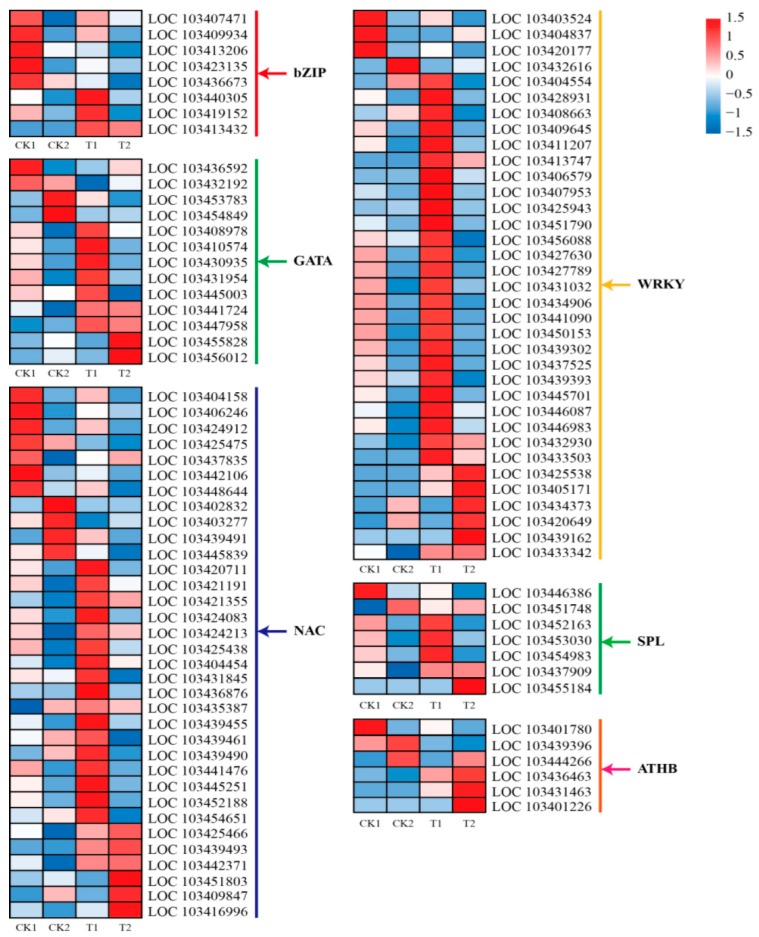
Heat map representation of the expression patterns of genes encoding transcription factors in “Granny Smith” apple skins. bZIP, basic region/leucine zipper motif; GATA, GATA zinc finger domain; NAC, NAM/ATAF/CUC; WRKY, protein domain containing a tryptophan-arginine-lysine-tyrosine amino acid motif; SPL, SQUAMOSA promoter-binding protein-like; ATHB, homeodomain leucine zipper proteins. Columns and rows in the heat map represent samples collected at different time points for which bags were removed. The color scale on the right represents the log-transformed FPKM value.

In our study, 114 putative TFs were altered in response to 5-aza-dC treatment and might regulate anthocyanin biosynthesis in bagged fruits of “Granny Smith”. In particular, three basic region/leucine zipper motifs (*bZIPs*), seven GATA zinc finger domains (*GATAs*), 17 NAM/ATAFs/CUCs (*NACs*), three SQUAMOSA promoter-binding protein-like members (*SPLs*), and 25 protein domains containing a tryptophan-arginine-lysine-tyrosine (*WRKY*) motifs were significantly increased in the T1 group, while two *GATAs*, six *NACs*, two *SPLs*, six *WRKYs*, and three homeodomain leucine zipper proteins (*ATHBs*) were significantly upregulated in fruits of the T2 group. In addition, five *bZIPs*, four *GATAs*, 11 *NACs*, two *SPLs*, four *WRKYs*, and three *ATHBs* were downregulated in response to 5-aza-dC treatment (Figure 5).

### 2.5. Expression of DNA Methylation-Related Genes

**Figure 6:**
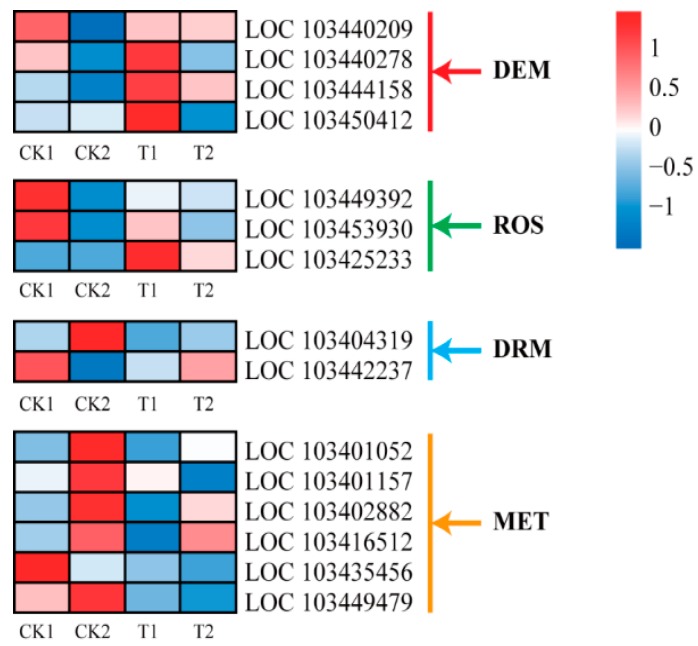
Heat map representation of expression patterns of methylase genes in “Granny Smith” apple skins. DME, demethylase; ROS, repressor of gene silencing; DRM, DNA (cytosine-5)-methyltransferase; MET, methyltransferase. Columns and rows in the heat map represent samples collected at different time points for which bags were removed. The color scale on the right represents the log-transformed FPKM value.

Expression levels of DNA methyltransferase (*MET* and *DRM*) and demethylase (*DME* and *ROS*) genes were investigated in response to 5-aza-dC treatment. Six *METs* and two *DRMs* were significantly downregulated in T groups compared with CK groups. Additionally, among demethylase genes, three *DMEs* and one *ROS* were slightly induced in T1 group (Figure 6). These findings suggest that the methylome pattern in apples could be dynamically altered by 5-aza-dC treatment.

### 2.6. Quantitative Real-Time PCR (qRT-PCR) Validation of DEGs

**Figure 7:**
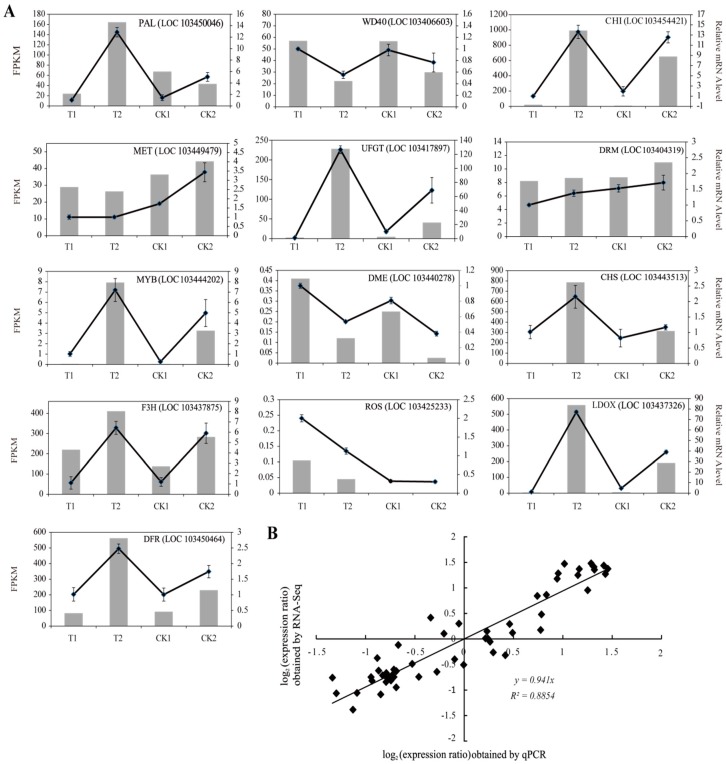
Quantitative real-time PCR (qRT-PCR) analysis of DEGs in “Granny Smith” apple skins. (**A**) Transcript levels and qRT-PCR results of 13 selected genes identified in RNA-sequencing. The left *y*-axis indicates the corresponding expression data from RNA-seq (gray histogram). The right *y*-axis shows the relative gene expression level measured by qRT-PCR (black lines). The *x*-axis represents the time (days) after 5-aza-dC treatment. Bars represent standard error (SE; *n* = 4). (**B**) Comparison between the log_2_ values of gene expression ratios obtained from RNA-seq and qRT-PCR methods.

To validate the reliability of the expression profiles obtained from RNA-seq, we selected 13 DEGs for qRT-PCR using specific primers to confirm the gene expression changes detected in the transcriptome analysis. These genes were *PAL*, *CHS*, *CHI*, *F3H*, *LDOX*, *UFGT*, and *DFR*, all involved in anthocyanin biosynthesis, *WD40* and *MYB1*, both of which regulate anthocyanin biosynthesis, and *MET*, *DRM*, *DME*, and *ROS* that are associated with DNA methylation. The exact fold change of DEGs at several data points varied between RNA-seq and qPCR methods (Figure 7A). However, the overall expression trends were strongly consistent (Pearson correlation coefficients *R*^2^ = 0.8854) (Figure 7B), confirming the reliability of the RNA-seq results.

## 3. Discussion

5-Aza-dC is an efficient inhibitor of DNA methylation [21] and is an ideal tool to uncover the phenomenon of red pigmentation in non-red apples. In the present work, we investigated the formation of red pigmentation in the skin of bagged “Granny Smith” apple following 5-aza-dC treatment. To our knowledge, this is the first report on the effects of 5-aza-dC on the pigmentation of apples based on transcriptome sequencing.

### 3.1. 5-Aza-dC Affects Anthocyanin Accumulation in the Skin of “Granny Smith” Apples

After 5-aza-dC treatment, the content of anthocyanins in apple skin was markedly increased (Figure 1B), suggesting that the enhanced red pigmentation was attributed to anthocyanin content. In particular, transcription of genes involved in the anthocyanin biosynthesis pathway was substantially increased in fruit skin following 5-aza-dC treatment (Figure 4). This indicates that 5-aza-dC promotes anthocyanin biosynthesis in “Granny Smith” apples after removal from bags. In addition, KEGG pathway analysis revealed that the most significantly enriched was flavonoid biosynthesis (Table 2), suggesting that 5-aza-dC had an important effect on apple color.

Anthocyanin biosynthesis is usually regulated by various TFs, especially the MBW complex [29]. Previously, *MYB* was found to be a crucial regulator of anthocyanin accumulation and fruit pigmentation in apples [30,31]. In the present study, 28 R2R3-MYB TFs were identified in skin samples of bagged “Granny Smith” apples following 5-aza-dC treatment, seven of which were significantly upregulated in transcription in the T2 group, corresponding to structural genes related to anthocyanin biosynthesis (Figure 4). These results indicate that these *MYBs* might be crucial regulators in the accumulation of anthocyanins in bagged “Granny Smith” following 5-aza-dC treatment. Additionally, the co-expression of *MYB* and *bHLH* was found to activate the expression of *DFR* and *UFGT*, leading to anthocyanin accumulation in nectarine (*Prunus persica*) [32]. In our study, seven *bHLH* genes were significantly upregulated in the T2 group, while the expression of other *bHLH* genes were upregulated in the T1 group of “Granny Smith” apples following 5-aza-dC treatment (Figure 4B). These results suggest that the *bHLH* family is involved in 5-aza-dC-induced anthocyanin synthesis through the different expression patterns.

### 3.2. Epigenetic Regulation of MYB in “Granny Smith” Apples Following 5-Aza-dC Treatment

The disruption of anthocyanin accumulation has been linked to changes in DNA methylation or demethylation in the promoter of the master anthocyanin regulator *MYB* [15,16,31]. Compared with “Honeycrisp” and “Royal Gala” [16], differences in methylation levels between control and 5-aza-dC-treated fruits were more obvious in “Granny Smith” [17], which reflects the striking contrast in pigmentation between the two fruits (i.e., an almost complete loss of anthocyanins in control fruits vs. red pigmentation in 5-aza-dC-treated fruits).

All three cytosine types (CG, CHG, and CHH) in the −1898 to −1633 bp region of the *MYB* (LOC103444202) promoter exhibited higher methylation levels in bagged “Granny Smith” apple skins after 5-aza-dC treatment (Supplementary Figure S1). Similar hypermethylation has also been observed in the three types of cytosines in the promoter of *PcMYB* in pears [15]. In the −2026 to −1870 bp region, methylation of two cytosine types (CHH and CHG) in fruit skins was dramatically lower in the former than the latter groups, but CG was not significantly different between 5-aza-dC-treated and control fruits (Supplementary Figure S1). This pattern of methylation in the −2026 to −1870 bp region appears to be rare, if not unique. Since the −2026 to −1870 bp region is located further upstream than the −1898 to −1633 bp region, and since methylation in the −2026 to −1870 bp region was correlated equally negatively with anthocyanin content and *MdMYB* expression, it would be interesting to investigate the methylation status in the −2026 to −1870 bp region more systematically in the future. Such work will further illustrate the epigenetic role of this type of regulation of *MYB* in apples.

### 3.3. Other TFs Related to Anthocyanin Biosynthesis Following 5-Aza-dC Treatment

Numerous other TFs exhibited differential expression in “Granny Smith” skins following 5-aza-dC treatment, especially some *bZIP*, *GATA*, *NAC*, *SPL*, *ATHB*, and *WRKY* genes. Thus, these TFs may function in anthocyanin biosynthesis in non-red apples induced by 5-aza-dC treatment. A recent report has suggested that a *bZIP* gene is involved in anthocyanin biosynthesis in apples [33]. Additionally, *GATA* TFs are important in plant growth and development, as well as the response to environmental stress [34]. In our study, the transcription of three *bZIPs* and nine *GATAs* was rapidly increased in bagged “Granny Smith” apples in response to 5-aza-dC treatment. This finding indicates that *bZIP* and *GATA* genes might be positive regulators of bagged “Granny Smith” apple pigmentation following 5-aza-dC treatment.

*NAC* participates in the modulation of anthocyanin biosynthesis by directly regulating *MYB* genes [35]. In this study, *NACs* were identified as the most abundant class of TFs upregulated by 5-aza-dC. This finding suggests that *NAC* may play important roles in apple pigmentation. One previous report revealed that *SPL* interacts with *MYB* in Chinese sand pear (*Pyrus pyrifolia*) [36]. In the current study, five *SPLs* were upregulated in treated fruits, suggesting that *SPL* is also involved in regulating anthocyanin biosynthesis in “Granny Smith” apples following 5-aza-dC treatment. Furthermore, *ATHBs* and *WRKYs* have also been reported to be involved in regulating anthocyanin biosynthesis in *Arabidopsis* and grapevine (*Vitis vinifera*), respectively [37,38]. Herein, although we identified many 5-aza-dC-induced or -suppressed TFs, the epigenetic regulation of anthocyanin biosynthesis in apples following 5-aza-dC treatment requires further investigation.

## 4. Materials and Methods

### 4.1. Plant Materials and Experimental Treatments

Fruits of the “Granny Smith” apple cultivar were collected at Baishui Apple Experimental Station, Northwest A & F University (35°21′ N, 109°55′ E, elevation 850 m; Yangling, China). Trees were grafted onto the M26 rootstock (*M. domestica*) and planted at a density of 4 m × 2 m. Young fruits were wrapped with two-layer paper bags (inner red, outer brown; Hong Tai, Xi’an, China) at 40 days after full bloom. Before the commercial harvest day, outer papers were removed at 160 days after full bloom. To protect bagged fruits from sunburn, inner papers were removed two days after outer papers. Fruits without the bagging treatment (unbagged fruits) were used as controls for comparison. Treatment of 5-aza-dC was applied according to Ma et al. [17]. Briefly, all fruits were divided into two groups, and one group was smeared with 1 mM 5-aza-dC (Sigma, St. Louis, MO, USA) plus 0.1% (*v*/*v*) Tween-20. The other group served as a control and was mock-treated with an equal volume of sterile distilled water. All smeared fruits were stored in a dark chamber (25 °C) for 24 h then exposed to white light (540 μmol·m^−2^·s^−1^) at 25 °C with a 16 h photoperiod. The 5-aza-dC smearing experiment was conducted before dark, alternating each day until completion. Apple skins were carefully harvested at six time points; 0, 2, 4, 6, 9, and 15 days after exposure of 5-aza-dC-treated fruits to light (days after 5-aza-dC treatment, DAFT). Fruit skin (~1 mm in thickness) was collected using an apple peeler according to the method described by Qu et al. [39]. The skins of candidate samples at 0 and 15 DAFT were subjected to transcriptome sequencing and expression analysis. All skins were immediately frozen in liquid nitrogen and stored at −80 °C until use.

### 4.2. Measurement of Anthocyanin and Chlorophyll Content

The measurement of anthocyanin content was performed as described previously [40]. Approximately 0.5 g apple skins were finely ground in 5 mL 1% (*v*/*v*) HCl-methanol for 24 h at 4 °C in darkness and centrifuged at 13,000× *g* for 10 min at 4 °C. Analysis was conducted on a high-performance liquid chromatography (HPLC) system equipped with a photo diode array (PDA) detector (Waters, Milford, MA, USA). Separation of anthocyanins was accomplished on a C18 column (5 μ, 250 mm × 4.6 mm internal diameter, Waters, USA) with cyanidin 3-galactoside as a standard (Sigma).

Chlorophyll was extracted with 80% acetone, and the content was determined on a UV-2550 ultraviolet spectrophotometer (Shimadzu Corp., Kyoto, Japan) according as previously described [41]. Four independent biological replicates were performed for each experiment. Data are expressed as means ± standard deviation and were assessed via one-way analysis of variance (ANOVA) followed by Tukey’s tests (*p* < 0.05) using SPSS 16.0 software (SPSS Inc., Chicago, IL, USA).

### 4.3. RNA Extraction, Library Preparation, and RNA-Seq

To ensure data reliability, two samples of each skin type were sequenced. Total RNA from triplicate samples was extracted using TRIzol RNA plant plus reagent (Tiangen, Beijing, China) according to the manufacturer’s protocol. RNA quality was assessed on an Agilent 2100 Bioanalyzer (Agilent Technologies, Palo Alto, CA, USA). Samples with an RNA integrity number (RIN) >7.5 were used for deep sequencing. Total mRNA was enriched and cleaved into small pieces for use as templates for cDNA synthesis, and cDNA fragments were purified with a QiaQuick PCR extraction kit (Qiagen, Venlo, The Netherlands), end-repaired, poly (A)-tailed, and ligated to Illumina sequencing adapters. After agarose gel electrophoresis, suitable products were used as templates for PCR amplification, and the resulting library was sequenced using an Illumina HiSeq^TM^ 2500 by Gene Denovo Biotechnology Co. (Guangzhou, China). In total, four sets of raw reads were obtained, corresponding to treatments at 0 DAFT (T1) and 15 DAFT (T2), and controls at 0 DAFT (CK1) and 15 DAFT (CK2).

### 4.4. RNA-Seq Data Processing and Mapping of Reads to the Apple Genome

Raw read processing of transcript datasets was performed by Gene Denovo Biotechnology Co., Ltd. (Guangzhou, China). To obtain high-quality clean reads, raw reads were further filtered by removing adapter sequences, poly-N read sequences, and low-quality sequences. All clean reads were mapped to the apple (*M*. *domestica*) genome [42] using SOA-Paligner/soap2 [43]. Clean reads were then aligned with the reference genome and transcripts were reconstructed with Cufflinks [44].

### 4.5. RNA-Seq Data Analysis

To confirm the accuracy and reliability of RNA-seq data, some poor-quality reads were eliminated from raw reads, and only remaining high-quality reads (clean reads) were used for statistical analysis. The level of gene expression was calculated using the Fragments Per Kilobase of transcript per Million (FPKM) mapped reads method [45]. DEGs between 5-aza-dC-treated and control groups were identified at the two different time points. Initial *p*-values were adjusted using the Benjamini and Hochberg approach [46] to minimize the false discovery rate (FDR). DEGs were assigned if the fold change was ≥2 and the FDR was <0.05. Functional annotation of DEGs was performed as described previously [47].

DEGs were subjected to Gene Ontology (GO) and Kyoto Encyclopedia of Genes and Genomes (KEGG) pathway enrichment analyses. For GO enrichment analysis, all DEGs were mapped to GO terms in the database (available online: http://www.geneontology.org/), and DAVID (available online: https://david.ncifcrf.gov/) was used to identify the main biological functions [48]. KEGG pathway enrichment analysis was performed using the appropriate database (available online: http://www.genome.jp/kegg/) to reveal significantly enriched metabolic and signal transduction pathways [49]. Significant GO/KEGG enrichment of DEGs comparing to the genome background was defined using hypergeometric tests. Calculated *p*-values were subjected to FDR correction using FDR ≤0.05 as a threshold.

### 4.6. RT-qPCR Validation

Total RNAs were reverse-transcribed to cDNAs using a PrimeScript Master Mix Kit (TaKaRa, Dalian, China) following the manufacturer’s instructions. Real-time PCR amplification and analysis was performed on an iQ5.0 instrument (Bio-Rad, Hercules, CA, USA) using a SYBR Premix Ex Taq Kit (TaKaRa) according to the manufacturer’s instructions. A two-step program was used, with an initial hot start at 95 °C for 30 s followed by 40 cycles at 95 °C for 5 s and 60 °C for 34 s. Melting curves were generated using the following program: 95 °C for 15 s, 60 °C for 1 min, and 95 °C for 15 s. The *actin* gene (GenBank: GQ339778.1) was used as an internal control for normalization. Specific primers were designed using Primer 5 software, and information is listed in Table S1. Data were analyzed according to the 2^−∆∆*C*T^ method [50]. Each reaction was performed in quadruplicate. Differences between means were analyzed using one-way ANOVA followed by post-hoc Tukey’s tests (*p* < 0.05). A *p*-value < 0.05 indicated a significant difference.

### 4.7. Data Accessibility

Sequencing data have been deposited in the NCBI Sequence Read Archive repository under accession numbers SAMN07972616, SAMN07972617, SAMN07972618, SAMN07972619, SAMN07972620, SAMN07972621, SAMN07972622, and SAMN07972623 (available online: https://www.ncbi.nlm.nih.gov/bioproject/416952), which will be released at 31 December 2018.

## 5. Conclusions

In this study, 5-aza-dC treatment enhanced red pigmentation in bagged fruits of “Granny Smith” apples. RNA sequencing revealed a total of 8482 DEGs between 5-aza-dC-treated and control groups of bagged fruits. After 5-aza-dC treatment, nearly all structural and regulatory genes such as *CHS*, *CHI*, *F3H*, *DFR*, *LDOX*, *UFGT*, *MYB* and *bHLH* involved in the anthocyanin biosynthetic pathway were upregulated. Additionally, *bZIP*, *GATA*, *NAC*, *SPL*, *WRKY*, and *ATHB* were more likely to have a tight connection with red pigmentation in bagged fruits following 5-aza-dC treatment. Furthermore, *METs*, *DRMs*, *DMEs*, and *ROSs* might be involved in 5-aza-dC-induced red pigmentation through different expression patterns. The transcriptome data and DEGs provide valuable information to unravel the regulatory mechanisms of 5-aza-dC-induced red pigmentation in bagged fruits of “Granny Smith” apples and possibly other non-red apple fruits.

## Figures and Tables

**Table 1:** Throughput and quality of RNA-seq of differentially expressed genes (DGEs) libraries from “Granny Smith” apple skins.

Sample	Clean Reads	Mapped Reads (%)	Uniquely Mapped Reads (%)	Expressed Genes
CK1-1	290,141,72	72.19	63.60	31713
CK1-2	250,432,60	70.62	62.39	29085
CK2-1	328,395,82	55.89	50.16	23434
CK2-2	295,493,66	64.48	57.98	26639
T1-1	238,889,26	70.18	61.79	30322
T1-2	270,839,02	71.39	62.91	31195
T2-1	319,614,16	72.05	64.33	30597
T2-2	315,852,92	71.33	63.55	30470

CK, control; T, 5-aza-2′-deoxycytidine treatment; 1-, 0 day after exposure of treated fruits to light; and 2-, 15 days after exposure of treated fruits to light; -1 and -2 represent replication 1 and 2, respectively.

**Table 2:** KEGG pathway enrichment analysis of DEGs in “Granny Smith” apple skins (*p* < 0.05).

Pathway	DEGs with Pathway Annotation (309)	All Genes with Pathway Annotation (10,222)	*p*-Value	Pathway ID
Flavonoid biosynthesis	12 (3.88%)	95 (0.93%)	0.000027	ko00941
Glutathione metabolism	17 (5.5%)	212 (2.07%)	0.000235	ko00480
Phenylpropanoid biosynthesis	25 (8.09%)	393 (3.84%)	0.000355	ko00940
Pyruvate metabolism	15 (4.85%)	210 (2.05%)	0.001767	ko00620
Phenylalanine metabolism	8 (2.59%)	80 (0.78%)	0.002791	ko00360
Aminoacyl-tRNA biosynthesis	11 (3.56%)	144 (1.41%)	0.004247	ko00970
Linoleic acid metabolism	5 (1.62%)	41 (0.4%)	0.007505	ko00591
Peroxisome	13 (4.21%)	200 (1.96%)	0.007693	ko04146
Fatty acid elongation	6 (1.94%)	60 (0.59%)	0.009244	ko00062
Carbon metabolism	28 (9.06%)	582 (5.69%)	0.009922	ko01200
Monoterpenoid biosynthesis	4 (1.29%)	30 (0.29%)	0.012073	ko00902
Nitrogen metabolism	6 (1.94%)	69 (0.68%)	0.017687	ko00910
Butanoate metabolism	4 (1.29%)	34 (0.33%)	0.018598	ko00650

## Supplementary Materials

Supplementary Materials can be found at http://www.mdpi.com/1422-0067/19/10/3133/s1.
