# Supplementary material for: Transcriptome Profiling Reveals Transcriptional Regulation by DNA Methyltransferase Inhibitor 5-Aza-2′-Deoxycytidine Enhancing Red Pigmentation in Bagged “Granny Smith” Apples (Malus domestica)
Source: Int J Mol Sci. 2018 Oct 12;19(10):3133. doi: 10.3390/ijms19103133 (PMC6213223; doi:10.3390/ijms19103133)
Supplement: Supplementary file 1 [file ijms-19-03133-s001.pdf]

Supplementary Materials: Transcriptome Profiling Reveals Transcriptional Regulation by DNA Methyltransferase Inhibitor 5-Aza-2′-Deoxycytidine Enhancing Red Pigmentation in Bagged "Granny Smith" Apples (*Malus domestica*)

Changqing Ma, Bowen Liang, Bo Chang, Li Liu, Jiuying Yan, Yazhou Yang and Zhengyang Zhao

Table S1. Primers used for quantitative real-time PCR in this work.

Gene ID	Name	Sense Primer (5′–3′)	Anti-Sense Primer (5′–3′)
LOC 103447714	ACTIN	CTGAACCCAAAGGCTAATCG	ACTGGCGTAGAGGGAAAGAA
LOC 103450046	PAL	AGGAACACCGTAAAGAACA	ACATACTCCCTATCGACAAC
LOC 103440278	DME	AGAATCCTGGGCTTGCTT	TCTCCATGCCTACGCTTT
LOC 103444202	MYB1	GTCGTCGTCAACAAAGAATGG	GGTCCGTGCTAAAGGAGAAT
LOC 103443513	CHS	AGTGACACCCACCTTGATAG	CTGTCGGGGAGAATGGTTTG
LOC 103437875	F3H	CTGCTACTACGCTGACATCC	AATACCCCAGTCCTCACAAG
LOC 103450464	DFR	ATTTATCTTTACGAGCATCC	CCCTATCTCCCTCAACTTCT
LOC 103437326	LDOX	GTTCCAAATTCCATCGTCAT	TCACCTTTTCCTTGTTCACC
LOC 103406603	WD40	AGAGCGGAGAACTCGGTGAC	CGATTCGGTGGTGCTGGTG
LOC 103454421	CHI	GTTACAGGTCCGTTTGAGAA	AACTTTTCAATGGCTTTGCCTTCTG
LOC 103417897	UFGT	TCGTAGCCTTCCCTTTCACT	TTATCAATGCTGTTGTTGGAAAAGA
LOC 103404319	DRM	GAGCAAACCAACCAGAG	ATCAAATCCGCCAAACT
LOC 103449479	MET	CGGAATAAGGGTGCTGAC	AGATACTCCTGGGAACGA
LOC 103425233	ROS	CGCATGAGCCAATTTAGGAC	CAAGAAGGTAAGGGCAGGGA

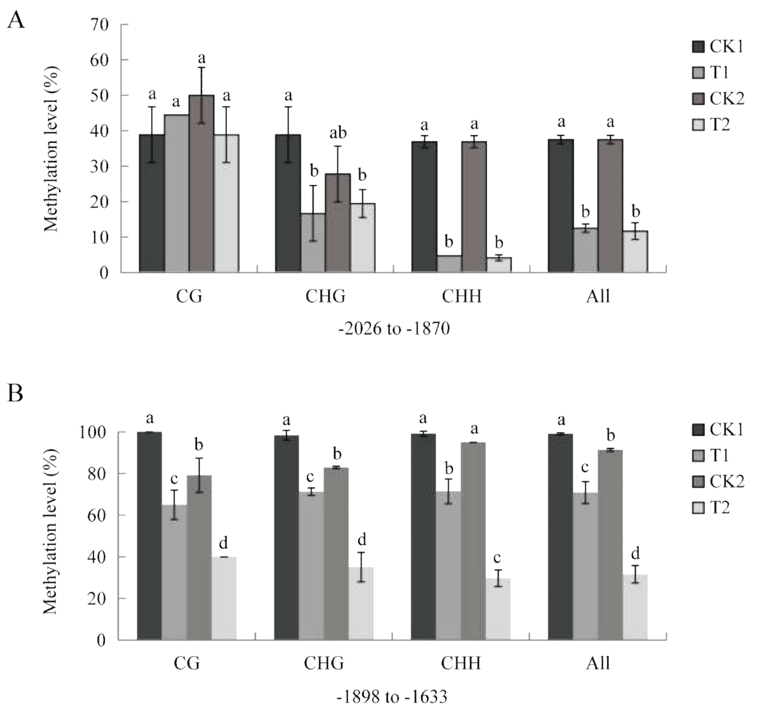

Figure S1. Bisulfite sequencing analysis of cytosine methylation levels in the *MYB* promoter in bagged "Granny Smith" apple skins of 5-aza-dC-treated and control fruits. The charts show the methylation levels of different cytosine types in the −2026 to −1870 (**A**) and −1898 to −1633 (**B**) regions. Twenty independent clones from each PCR were sequenced and analyzed using the Kismeth software. In the *x*-axis, "All" refers to overall methylated cytosines, while CG, CHG, and CHH refer to the three different types, in which H represents A, C, or T nucleotides. Different lower case letters indicate significant differences by Tukey's multiple range test ($p < 0.05$).
